# Supplementary material for: Eight years of community structure monitoring through recreational citizen science at the “SS Thistlegorm” wreck (Red Sea)
Source: PLoS One. 2023 Mar 15;18(3):e0282239. doi: 10.1371/journal.pone.0282239 (PMC10016724; doi:10.1371/journal.pone.0282239)
Supplement: S1 Table — * Indicate significant differences (P < 0.05). (DOCX) [file pone.0282239.s001.docx]

**S1 Table. Yearly sample statistics of the Relate test and significance of the relation between taxa data sets (abundance and presence/absence) and diving parameters.** * Indicate significant differences (*P* < 0.05).

| **Year** | **Rho** | **Significance Level (%)** |
| --- | --- | --- |
| **Abundance data** | | |
| 2007 | 0.162 | 0.7* |
| 2008 | 0.157 | 0.3* |
| 2009 | 0.111 | 4.6* |
| 2010 | 0.009 | 42.6 |
| 2011 | 0.104 | 6.9 |
| 2012 | 0.217 | 1* |
| 2013 | 0.242 | 6.4 |
| 2014 | -0.128 | 70.8 |
| **Presence/Absence data** | | |
| 2007 | 0.18 | 0.2* |
| 2008 | 0.136 | 1.4* |
| 2009 | 0.106 | 6.3 |
| 2010 | 0.015 | 40.7 |
| 2011 | 0.079 | 15 |
| 2012 | 0.209 | 0.5* |
| 2013 | 0.194 | 10.4 |
| 2014 | -0.097 | 64.4 |
