# Supplementary material for: Eight years of community structure monitoring through recreational citizen science at the “SS Thistlegorm” wreck (Red Sea)
Source: PLoS One. 2023 Mar 15;18(3):e0282239. doi: 10.1371/journal.pone.0282239 (PMC10016724; doi:10.1371/journal.pone.0282239)
Supplement: S2 Table — * Indicates significant relationship (P < 0.05). (DOCX) [file pone.0282239.s002.docx]

**S2 Table. DistLM sequential test table of results for each year with a significant Relate test between taxa abundance data and diving parameters data.** * Indicates significant relationship (*P* < 0.05).

| **Variable** | **R^2** | **SS(trace)** | **Pseudo-F** | **P** |
| --- | --- | --- | --- | --- |
| **2007** | | | | |
| Percentage of Year | 3.24E-02 | 7139.6 | 2.7418 | 0.001* |
| Maximum Depth | 5.24E-02 | 4424.3 | 1.7139 | 0.043* |
| Depth with Most Time Spent | 8.08E-02 | 6259.6 | 2.4688 | 0.005* |
| Water Temperature | 0.11721 | 8040.8 | 3.2609 | 0.001* |
| Dive Duration | 0.1455 | 6242.9 | 2.5825 | 0.002* |
| Fraction of Day | 0.17072 | 5565.3 | 2.3418 | 0.003* |
| **2008** | | | | |
| Percentage of Year | 6.59E-02 | 10516 | 4.2355 | 0.001* |
| Maximum Depth | 0.10501 | 6231.5 | 2.5756 | 0.004* |
| Depth with Most Time Spent | 0.13201 | 4307.2 | 1.8046 | 0.033* |
| Water Temperature | 0.16697 | 5575.7 | 2.3921 | 0.008* |
| Dive Duration | 0.19716 | 4814 | 2.1053 | 0.017* |
| Fraction of Day | 0.21578 | 2970.5 | 1.3062 | 0.195 |
| **2009** | | | | |
| Percentage of Year | 6.48E-02 | 9698.4 | 3.9507 | 0.001* |
| Maximum Depth | 9.06E-02 | 3853.3 | 1.5858 | 0.102 |
| Depth with Most Time Spent | 0.13677 | 6912.2 | 2.9434 | 0.001* |
| Water Temperature | 0.16299 | 3923.3 | 1.6917 | 0.053 |
| Dive Duration | 0.18001 | 2545.9 | 1.0998 | 0.329 |
| Fraction of Day | 0.22789 | 7165.5 | 3.2253 | 0.001* |
| **2012** | | | | |
| Percentage of Year | 0.10713 | 7681 | 3.2394 | 0.001* |
| Maximum Depth | 0.15342 | 3319.4 | 1.4218 | 0.15 |
| Depth with Most Time Spent | 0.18313 | 2130.4 | 0.90932 | 0.549 |
| Water Temperature | 0.2546 | 5124.6 | 2.3012 | 0.004* |
| Dive Duration | 0.30535 | 3638.6 | 1.6802 | 0.077 |
| Fraction of Day | 0.34348 | 2733.9 | 1.2777 | 0.239 |
