# Supplementary material for: Eight years of community structure monitoring through recreational citizen science at the “SS Thistlegorm” wreck (Red Sea)
Source: PLoS One. 2023 Mar 15;18(3):e0282239. doi: 10.1371/journal.pone.0282239 (PMC10016724; doi:10.1371/journal.pone.0282239)
Supplement: S3 Table — * Indicates significant relationship (P < 0.05). (DOCX) [file pone.0282239.s003.docx]

**S3 Table.** **DistLM sequential test table of results for each year with a significant Relate test between taxa presence/absence data and diving parameters data.** * Indicates significant relationship (*P* < 0.05).

| **Variable** | **R^2** | **SS(trace)** | **Pseudo-F** | **P** |
| --- | --- | --- | --- | --- |
| **2007** | | | | |
| Percentage of Year | 3.19E-02 | 6025.3 | 2.6996 | 0.004* |
| Maximum Depth | 5.01E-02 | 3448.6 | 1.5556 | 0.117 |
| Depth with Most Time Spent | 7.84E-02 | 5346.3 | 2.4549 | 0.007* |
| Water Temperature | 0.11628 | 7161.3 | 3.3865 | 0.001* |
| Dive Duration | 0.14611 | 5640.3 | 2.7254 | 0.003* |
| Fraction of Day | 0.16696 | 3940.6 | 1.9268 | 0.032* |
| **2008** | | | | |
| Percentage of Year | 5.11E-02 | 6812.9 | 3.2286 | 0.001* |
| Maximum Depth | 8.97E-02 | 5156 | 2.5047 | 0.004* |
| Depth with Most Time Spent | 0.11369 | 3199.3 | 1.5691 | 0.114 |
| Water Temperature | 0.15081 | 4953.1 | 2.4918 | 0.007* |
| Dive Duration | 0.1813 | 4068.6 | 2.0858 | 0.031* |
| Fraction of Day | 0.20006 | 2502.8 | 1.2897 | 0.238 |
| **2012** | | | | |
| Percentage of Year | 9.76E-02 | 6197.1 | 2.9202 | 0.003* |
| Maximum Depth | 0.15262 | 3493.6 | 1.6882 | 0.087 |
| Depth with Most Time Spent | 0.17632 | 1505 | 0.71941 | 0.703 |
| Water Temperature | 0.25321 | 4882.3 | 2.4711 | 0.015* |
| Dive Duration | 0.3159 | 3980 | 2.1074 | 0.049* |
| Fraction of Day | 0.34834 | 2060.1 | 1.0953 | 0.375 |
