# Supplementary material for: Eight years of community structure monitoring through recreational citizen science at the “SS Thistlegorm” wreck (Red Sea)
Source: PLoS One. 2023 Mar 15;18(3):e0282239. doi: 10.1371/journal.pone.0282239 (PMC10016724; doi:10.1371/journal.pone.0282239)
Supplement: S4 Table — PY as date of dive (expressed as percentage of year), MD as maximum depth, AD as average depth, T as temperature, RTD as dive time, and FD as hour (expressed as percentage of day). (DOCX) [file pone.0282239.s004.docx]

**S4 Table. Yearly averages of all diving parameters with confidence intervals.** PY as date of dive (expressed as percentage of year), MD as maximum depth, AD as average depth, T as temperature, RTD as dive time, and FD as hour (expressed as percentage of day).

| **Year** | **Average** | **Confidence Interval** |
| --- | --- | --- |
| **PY (% of Year)** | | |
| **2007** | 56.3 | 50.9-61.7 |
| **2008** | 73.4 | 71.2-75.6 |
| **2009** | 81.6 | 80.1-83.1 |
| **2010** | 62.4 | 58.9-67.7 |
| **2011** | 74.5 | 69.9-79.1 |
| **2012** | 52.7 | 46.5-58.9 |
| **2013** | 61.6 | 60.4-62.8 |
| **2014** | 35.3 | 35.4-35.3 |
| **MD (m)** | | |
| **2007** | 27.3 | 26.8-27.8 |
| **2008** | 27.1 | 26.4-27.7 |
| **2009** | 28.4 | 27.7-29.0 |
| **2010** | 28.4 | 27.9-28.9 |
| **2011** | 27.1 | 26.2-28.0 |
| **2012** | 26.4 | 25.4-27.3 |
| **2013** | 28.9 | 26.8-31.1 |
| **2014** | 27.5 | 26.2-28.7 |
| **AD (m)** | | |
| **2007** | 18.9 | 18.3-19.5 |
| **2008** | 19.3 | 18.5-20.0 |
| **2009** | 20.3 | 19.5-21.2 |
| **2010** | 22.3 | 21.5-23.2 |
| **2011** | 19.5 | 18.7-20.3 |
| **2012** | 20.0 | 18.6-21.3 |
| **2013** | 19.6 | 18.0-21.1 |
| **2014** | 20.1 | 18.3-21.8 |
| **T (^o^C)** | | |
| **2007** | 25.3 | 24.8-25.8 |
| **2008** | 26.0 | 25.7-26.3 |
| **2009** | 26.3 | 26.2-26.5 |
| **2010** | 26.6 | 25.2-27.1 |
| **2011** | 24.8 | 24.5-25.1 |
| **2012** | 25.7 | 24.8-26.6 |
| **2013** | 27.5 | 27.1-27.9 |
| **2014** | 23.9 | 23.3-24.5 |
| **RTD (mins)** | | |
| **2007** | 44.2 | 43.2-45.2 |
| **2008** | 44.8 | 43.4-46.2 |
| **2009** | 41.2 | 39.7-42.9 |
| **2010** | 49.9 | 47.5-52.4 |
| **2011** | 45.7 | 44.6-46.7 |
| **2012** | 43.5 | 41.8-45.3 |
| **2013** | 47.3 | 45.0-49.7 |
| **2014** | 54.8 | 51.8-57.7 |
| **FD (% of Day)** | | |
| **2007** | 0.50 | 0.48-0.52 |
| **2008** | 0.46 | 0.45-0.48 |
| **2009** | 0.48 | 0.46-0.49 |
| **2010** | 0.46 | 0.43-0.48 |
| **2011** | 0.46 | 0.45-0.48 |
| **2012** | 0.46 | 0.44-0.48 |
| **2013** | 0.49 | 0.45-0.52 |
| **2014** | 0.49 | 0.46-0.51 |
