# Supplementary material for: Eight years of community structure monitoring through recreational citizen science at the “SS Thistlegorm” wreck (Red Sea)
Source: PLoS One. 2023 Mar 15;18(3):e0282239. doi: 10.1371/journal.pone.0282239 (PMC10016724; doi:10.1371/journal.pone.0282239)
Supplement: Attachment — Submitted filename: Peirano short letter to Editor and Authos.pdf [file pone.0282239.s005.pdf]

Referee to:

Eight years of community structure monitoring through recreational citizen science atthe "SS Thistlegorm" wreck (Red Sea)
Manuscript Number: PONE-D-22-

The work is very interesting and should be published as one of the first paper on the use of citizen science for monitoring the environment.

I recommend a minor review of the ms mainly in term of presentation. I remember to the authors that scientific paper should give the possibility to the readers to replicate the methods in other sites of the words.

There is an huge amount of data to support the ms. I cannot download the annexes, however, I think that the authors should have the patience to describe and show methods and data in a more detailed way. I think that at last they will be satisfied of the final result.

The detailed comments and suggestions are in the in the .pdf file.

Good work and compliments,

Andrea Peirano
