# Supplementary material for: Eight years of community structure monitoring through recreational citizen science at the “SS Thistlegorm” wreck (Red Sea)
Source: PLoS One. 2023 Mar 15;18(3):e0282239. doi: 10.1371/journal.pone.0282239 (PMC10016724; doi:10.1371/journal.pone.0282239)
Supplement: Attachment — Submitted filename: PONE-D-22-26446_reviewer.pdf [file pone.0282239.s007.pdf]

PLOS ONE

Eight years of community structure monitoring through recreational citizen science at the “SS Thistlegorm” wreck (Red Sea)

--Manuscript Draft--

**Manuscript Number:**	PONE-D-22-26446
**Article Type:**	Research Article
**Full Title:**	Eight years of community structure monitoring through recreational citizen science at the “SS Thistlegorm” wreck (Red Sea)
**Short Title:**	Community structure at the Thistegorm wreck
**Corresponding Author:**	Erik Caroselli<br>University of Bologna<br>Bologna, ITALY
**Keywords:**	biodiversity; wreck dive; artificial reef; coral reefs
**Abstract:**	Large artificial coral reef communities, such as those thriving on sunken shipwrecks, tend to mirror those of nearby natural coral reefs and their long-term dynamics may help future reef resilience to environmental change. We examined the biodiversity of the world-renown “SS Thistlegorm” wreck in the northern Red Sea from 2007 through 2014, analyzing data collected during the recreational citizen science Red Sea monitoring project “Scuba Tourism for the Environment” (www.steproject.org). Volunteer divers collected data about date of the dive, maximum depth, average depth, temperature, dive time, hour of dive, and gave an abundance estimation of sighted taxa from a list of 72 target taxa. Although yearly variations in biodiversity were significant, there was no clear temporal trend in community variation, and biodiversity remained high throughout the 8 years (71 of all 72 target taxa were sighted). The 5 main taxa driving variations among year clusters in presence/absence and abundance taxa data were determined. The well-established community structure at the “SS Thistlegorm” provides a compelling example of how artificial coral reefs can sustain a stable and relatively similar biodiversity as their natural counterparts.
**Order of Authors:**	Chloe Lee
	Erik Caroselli
	Mariana Machado Toffolo
	arianna mancuso
	Chiara Marchini
	Marta Meschini
	Stefano Goffredo
**Additional Information:**	
**Question**	**Response**
**Financial Disclosure**<br><br>Enter a financial disclosure statement that describes the sources of funding for the work included in this submission. Review the submission guidelines for detailed requirements. View published research articles from *PLOS ONE* for specific examples.<br><br>This statement is required for submission and **will appear in the published article** if	STE project was funded by Project AWARE Foundation, ASTOI Association, Ministry of Tourism of the Arab Republic of Egypt, Settemari S.p.A Tour Operator, Scuba Nitrox Safety International, Viaggio nel Blu Diving Center

Powered by Editorial Manager® and ProduXion Manager® from Aries Systems Corporation

the submission is accepted. Please make sure it is accurate.<br><br>**Unfunded studies**<br>Enter: *The author(s) received no specific funding for this work.*<br><br>**Funded studies**<br>Enter a statement with the following details:<br>• Initials of the authors who received each award<br>• Grant numbers awarded to each author<br>• The full name of each funder<br>• URL of each funder website<br>• Did the sponsors or funders play any role in the study design, data collection and analysis, decision to publish, or preparation of the manuscript?<br>• **NO** - Include this sentence at the end of your statement: *The funders had no role in study design, data collection and analysis, decision to publish, or preparation of the manuscript.*<br>• **YES** - Specify the role(s) played.<br><br>* typeset	
**Competing Interests**<br><br>Use the instructions below to enter a competing interest statement for this submission. On behalf of all authors, disclose any competing interests that could be perceived to bias this work—acknowledging all financial support and any other relevant financial or non-financial competing interests.<br><br>This statement is required for submission and **will appear in the published article** if the submission is accepted. Please make sure it is accurate and that any funding sources listed in your Funding Information later in the submission form are also declared in your Financial Disclosure statement.<br><br>View published research articles from *PLOS ONE* for specific examples.	NO authors have competing interests

**NO authors have competing interests**<br><br>Enter: *The authors have declared that no competing interests exist.*<br><br>**Authors with competing interests**<br><br>Enter competing interest details beginning with this statement:<br><br>*I have read the journal's policy and the authors of this manuscript have the following competing interests: [insert competing interests here]*<br><br>* typeset	
**Ethics Statement**<br><br>Enter an ethics statement for this submission. This statement is required if the study involved:<br><br>• Human participants<br>• Human specimens or tissue<br>• Vertebrate animals or cephalopods<br>• Vertebrate embryos or tissues<br>• Field research<br><br>Write "N/A" if the submission does not require an ethics statement.<br><br>General guidance is provided below. Consult the submission guidelines for detailed instructions. **Make sure that all information entered here is included in the Methods section of the manuscript.**	Participants (or parents/guardians in case of minors) gave their consent by signing a declaration inserted in the questionnaires. STEproject and its consent acquisition procedure have<br>received the approval of Bioethics Committee of the University of Bologna (prot. 2.6).

Powered by Editorial Manager® and ProduXion Manager® from Aries Systems Corporation

**Format for specific study types**<br><br>**Human Subject Research (involving human participants and/or tissue)**<br>• Give the name of the institutional review board or ethics committee that approved the study<br>• Include the approval number and/or a statement indicating approval of this research<br>• Indicate the form of consent obtained (written/oral) or the reason that consent was not obtained (e.g. the data were analyzed anonymously)<br><br>**Animal Research (involving vertebrate animals, embryos or tissues)**<br>• Provide the name of the Institutional Animal Care and Use Committee (IACUC) or other relevant ethics board that reviewed the study protocol, and indicate whether they approved this research or granted a formal waiver of ethical approval<br>• Include an approval number if one was obtained<br>• If the study involved *non-human primates*, add *additional details* about animal welfare and steps taken to ameliorate suffering<br>• If anesthesia, euthanasia, or any kind of animal sacrifice is part of the study, include briefly which substances and/or methods were applied<br><br>**Field Research**<br><br>Include the following details if this study involves the collection of plant, animal, or other materials from a natural setting:<br>• Field permit number<br>• Name of the institution or relevant body that granted permission	
**Data Availability**<br><br>Authors are required to make all data underlying the findings described fully available, without restriction, and from the time of publication. PLOS allows rare exceptions to address legal and ethical concerns. See the PLOS Data Policy and FAQ for detailed information.	Yes - all data are fully available without restriction

Powered by Editorial Manager® and ProduXion Manager® from Aries Systems Corporation

A Data Availability Statement describing where the data can be found is required at submission. Your answers to this question constitute the Data Availability Statement and **will be published in the article**, if accepted.<br><br>**Important:** Stating 'data available on request from the author' is not sufficient. If your data are only available upon request, select 'No' for the first question and explain your exceptional situation in the text box.<br><br>Do the authors confirm that all data underlying the findings described in their manuscript are fully available without restriction?	
**Describe where the data may be found in full sentences. If you are copying our sample text, replace any instances of XXX with the appropriate details.**<br><br>• If the data are **held or will be held in a public repository**, include URLs, accession numbers or DOIs. If this information will only be available after acceptance, indicate this by ticking the box below. For example: *All XXX files are available from the XXX database (accession number(s) XXX, XXX.*).<br>• If the data are all contained **within the manuscript and/or Supporting Information files**, enter the following: *All relevant data are within the manuscript and its Supporting Information files.*<br>• If neither of these applies but you are able to provide **details of access elsewhere**, with or without limitations, please do so. For example:<br><br>*Data cannot be shared publicly because of [XXX]. Data are available from the XXX Institutional Data Access / Ethics Committee (contact via XXX) for researchers who meet the criteria for access to confidential data.*<br><br>*The data underlying the results presented in the study are available from (include the name of the third party*	Data are stored in figshare under this doi: 10.6084/m9.figshare.21197269. The data will be made publicly available upon publication

and contact information or URL).<br>• This text is appropriate if the data are owned by a third party and authors do not have permission to share the data.<br><br>* typeset	
Additional data availability information:	Tick here if the URLs/accession numbers/DOIs will be available only after acceptance of the manuscript for publication so that we can ensure their inclusion before publication.

Powered by Editorial Manager® and ProduXion Manager® from Aries Systems Corporation

Manuscript

Click here to access/download;Manuscript;PLoS one Draft.docx

Eight years of community structure monitoring through recreational citizen science at the "SS Thistlegorm" wreck (Red Sea)

Chloe Lee[1], Erik Caroselli[1,2*], Mariana Machado Toffolo[1,2], Arianna Mancuso[1,2], Chiara Marchini[1,2], Marta Meschini[1,2], Stefano Goffredo[1,2]

[1] Marine Science Group, Department of Biological, Geological and Environmental Sciences, University of Bologna, Via Selmi 3, 40126 Bologna (Italy)

[2] Fano Marine Center, The Inter-Institute Center for Research on Marine Biodiversity, Resources and Biotechnologies, Viale Adriatico 1/N, 61032 Fano, Italy

* Corresponding author: Erik Caroselli, erik.caroselli@unibo.it, +39 051 2094157

Keywords: biodiversity; wreck dive; artificial reef; coral reefs

Abstract

Large artificial coral reef communities, such as those thriving on sunken shipwrecks, tend to mirror those of nearby natural coral reefs and their long-term dynamics may help future reef resilience to environmental change. We examined the biodiversity of the world-renown "SS Thistlegorm" wreck in the northern Red Sea from 2007 through 2014, analyzing data collected during the recreational citizen science Red Sea monitoring project "Scuba Tourism for the Environment" (www.steproject.org). Volunteer divers collected data about date of the dive, maximum depth, average depth, temperature, dive time, hour of dive, and gave an abundance estimation of sighted taxa from a list of 72 target taxa. Although yearly variations in biodiversity were significant, there was no clear temporal trend in community variation, and biodiversity remained high throughout the 8 years (71 of all 72 target taxa were sighted). The 5 main taxa driving variations among year clusters in presence/absence and abundance taxa data were determined. The well-established community structure at the "SS Thistlegorm" provides a compelling example of how artificial coral reefs can sustain a stable and relatively similar biodiversity as their natural counterparts.

Introduction

The biological communities of tropical and sub-tropical coral reefs support some of the highest biodiversity in the world [1] and provide a wide array of socio-economic services including coastal protection, water quality and chemical cycling, fisheries and materials markets, and experiential benefits [2]. Despite their assortment of values, coral species homogenization [3], species extinctions [4], and overall changes of coral communities are expected in future [5]. In fact, the Intergovernmental Panel on Climate Change projections [6] and current research indicate a multitude of threats to coral reefs including but not limited to ocean warming and acidification, overfishing, and rise in coastal human populations and impacts [7].

One of the largest coral reef systems of the world, with notably high endemism, lies within the Red Sea [8]. Due to its peculiar geo-evolutionary past resulting in large latitudinal gradients of sea surface temperature and salinity [8] the Red Sea provides an attractive location for studying the response of coral reefs to threats of their projected immediate future. The northern Red Sea is considered a coral refuge because, although it has experienced some of the highest sea surface temperature anomalies, there have been very few coral bleaching events compared to its southern portion [7,9]. Notwithstanding the relative resilience of reefs in the northern Red Sea, there is still evidence of general coral colony size decline and species homogenization throughout [10], having the potential to induce changes in the biological communities they support. The importance of monitoring community variation lies not only in its fundamental value but also the economic income from fisheries [11] and especially the tourist industry, providing an array of socio-economic opportunities for many coastal countries [12]. Unfortunately, there is general lack of information regarding Red Sea coral communities, which are relatively understudied when compared to the Great Barrier Reef or reef systems of the Caribbean Sea [13]. This can pose conservation challenges when attempting to monitor and analyze crucial changes in the biological communities of the Red Sea.

An innovative and upcoming approach to gathering large amounts of data in a time efficient way is the use of citizen science. This approach is not so novel in terms of human history,

but current technological advances of citizen science in the spreading and sharing of information and data [14,15] have benefits and uses that are now an integral part of modern science [16]. Citizen science can be a critical asset in helping researchers, policy makers, and stakeholders overcome resource limitations (e.g., economical, temporal, geographical). Community-based management, and community-based monitoring are two of the main methodologies of the citizen science approach. The former involves direct participation of citizens and stakeholders in management decisions [17], while the latter implies the collaboration of citizens and stakeholder for data collection to monitor, track, and respond to areas of concern (e.g., environmental health) [18]. Scuba Tourism for the Environment (STE; www.steproject.org) has been a great example of utilizing large numbers of volunteers for community-based monitoring throughout much of the Red Sea [19]. Project volunteers participating in the STE program collected ecological community data with the assistance of active dive centers of their choosing and trained guides throughout Egypt. Recreational citizen science was applied throughout the project and therefore did not require a behavioral change of the divers. Reliability trials of the STE project, based on correlations to reference researchers, indicate that Consistency ranked the lowest parameter while Percent Identified scored the highest [20], showing that while divers can accurately identify most taxa there is a tendency to focus on certain organisms due to personal interest. Overall, with correct development to the needs of specific projects, data collected through citizen science can reliably support monitoring efforts [20, 17,16]. The SS Thistlegorm, a world class dive site [21] and one of topmost visited wreck dives in the world [22], was one of the dive locations monitored in the STE project and is the focus of this study.

Built in 1940, the SS Thistlegorm was quickly taken over for the war efforts of the British military and after 3 successful voyages, her fourth and final incomplete journey from Glasgow (UK) to Alexandria (Egypt) resulted in her ultimate resting ground in the Straights of Gubal on the 6th of October 1941, 30 meters below the sea surface [23]. Following two hits by German Heinkel HE 111 bombers to the fourth hold and the engine room (where the ammunition was stored), the 128 m long cargo steam ship was split in two by munitions explosions and sunk within minutes. In the 80 plus

years since the subsequent discovery of the wreck by Jacques-Yves Cousteau in 1955, and the development of the Sharm el Sheikh resort in the 1990s, the SS Thistlegorm has recruited up to 175,000 visiting divers per year from around the world [23]. Due to its high popularity and frequent visitors, there are rising concerns regarding the structural longevity of this historic site. Irresponsibly moored boats, inexperienced divers, and even air bubbles can cause irreparable damage to the structural integrity of underwater relics [24] as well as the biological community residing there [25]. A dedicated team of divers, archeologists, and researchers developed the Thistlegorm Project (www.thethistlegormproject.com) to survey the site using 360 videography for the purposes of creating an accurate archeological 3D survey and to raise awareness for the protection of underwater cultural heritage sites.

Aside from the historical importance of the SS Thistlegorm wreck, the ship has recruited numerous marine life forms through the decades spent on the sandy bottom close to a natural coral reef and far from touristic resorts. In fact, this underwater, man-made structure can be regarded as a an artificial reef community, though “accidental” in nature. Since the 1960’s, artificial reefs have been used for a variety of purposes including biodiversity conservation [26], fisheries management [27], and tourist locations [28]. One study in Eilat (Red Sea) designed and submerged multiple concrete structures to test whether artificial reefs could decrease diving pressures on the surrounding natural reef areas, concluding that small scale structures, knowledge of such structures within the local diving community, and education of divers could be sufficient [29]. The provision of migratory networks for coral movement and resettlement within changing oceans [30] is another application for artificial reefs, though highly debated and rather unexplored. Organismal community recruitment and interchange is an evident and well observed occurrence when any new colonizable object, be it an island or a plastic bottle, appears near a source population. In accordance with island biogeography theory [31], the evolution of this community is much dependent on the size of the object in question, the time it is in existence, and its distance from the source. Sunken warships have been shown to serve as exceptionally good artificial substrates for coral reefs as their size and complexity offer a

multitude of opportunities for microhabitats, and in cases where they extend down to 30-40 m, the cooler waters can provide respite to corals from warming oceans but still sufficient light for their symbiotic zooxanthellae [32]. Many shipwrecks in the North Sea have supported hard substrate communities throughout the Belgian Continental Shelf which has otherwise been transformed into a soft-bottom environment due to anthropogenic activity [33, 34[illegible]hanks to the steel structure of the SS Thistlegorm, recruitment of various tolerant sessile species typical of hard substrates was enabled in the sandy bottom environment within the Straights of Gubal and a well-established coral community can now be witnessed, but no information is available in the scientific literature.

The aim of this study was to characterize the community structure of one of the most well-known and top-raking dive sites in the world, the SS Thistlegorm, over 8 years of monitoring through recreational citizen science.

Materials and methods

Data Collection and Isolation

Data were collected within the Scuba Tourism for the Environment - Red Sea Biodiversity Monitoring Program (STE; www.steproject.org), whose efforts include the collection of large scale spatial-temporal biodiversity data within the Red Sea from 2007 to 2015 using a recreational citizen science approach where the behavior of the underwater tourists is unaltered during surveys [19]. Participant volunteers completed questionnaires that contained general demographic information (name, country of residence, address, diving certification, etc.), dive site and environment type (sandy bottom, wreck, blue, or coral reef), six diving parameters (date, maximum depth, depth where they spent most of their time, temperature, dive time, and hour), and the sightings of 72 target taxa which were chosen due to easier recognition (for the detailed methods see 19). Data coming from the Thistlegorm wreck site (27° 48’ 30.59’’ N and 33° 55’ 7.19’’ E; Figure 1) were isolated from the compiled STE data set and aggregated per year. Nine questionnaires from 2015 were removed from the analysis, as per lack of efficacy for less than 10 questionnaires per year (following 19). While Branchini and colleagues [19] examined the “coral reef” environment questionnaires, this study

reports findings from an unpublished data set within the "wreck" environment type. Data from a total of 390 questionnaires between 2007 and 2014 were included in this analysis (Table 1).

Figure 1. Map of data collection site within the Red Sea. The orange star indicates the approximate location of the SS Thistlegorm wreck.

Table 1. Yearly counts of completed questionnaires.

Year	**2007**	**2008**	**2009**	**2010**	**2011**	**2012**	**2013**	**2014**	**Total**
Number of Questionnaires	84	62	59	59	72	29	12	13	390

Preliminary Treatment and Analyses

Data were split into three data sets: 1) taxa abundance; 2) taxa presence/absence (p/a), obtained through an overall transformation of taxa abundance; and 3) diving parameters. The sighting frequency (*SF%*) and Relative Abundance (*RA*) for each taxon were calculated as such [19]:

$$SF\% = \left(\frac{Number\ of\ dives}{number\ of\ sightings\ per\ taxon}\right)\ x\ 100$$

$$RA = (SF\%)(Average\ Sighting\ Abundance)$$

For a general picture of the community composition and to identify outliers within each year, 2-dimensional plots were created using non-metric multidimensional scaling (MDS) from the Bray-Curtis resemblance matrices of both taxa data sets [35, 36]. A BVSTEP "Best" test was performed on both taxa data sets to find the subset of taxa that best represented the community of each year (BVSTEP [37]). These subsets of taxa were later used for aggregation of the data in the temporal analysis

A distance matrix of normalized diving parameters was created using Euclidean distances. Both taxa distance matrices were tested for a relationship with the distance matrices of the diving parameters using the Relate test to determine whether the variation between years was affected only by the change in time or also by diving parameters (Relate, [38]). Additional distance based linear models (DistLM, [39]) were ran for each year with a significant relationship between the taxa data

and diving parameters, providing the individual parameters that significantly affected the variation of community structure in each year.

To test the differences in diving parameters over the years, a permutational multivariate analysis of variance (PERMANOVA, [39]) was designed with the fixed factor of "year" and run on the Euclidean resemblance matrix of the normalized aggregate diving parameters. Both main and pairwise tests were run. Additional PERMANOVAs were created for individual diving parameters to test whether each different diving parameter was significantly different among the years.

All the above analyses were done using PRIMER-E version 6 with PERMANOVA+ version 1 software (PRIMER-E, Ltd., Ivybridge, UK).

Seven of the dives within the entire dataset were viable for validation trials and were analyzed to measure the quality of volunteer collected data. The mean similarity index, accuracy, and correctness of abundance ratings (CAR) parameters were tested in accordance with the standard methodology for this citizen science project [20].

Temporal Analysis

Two reduced datasets containing only the best subsets of taxa from any individual year were obtained, and resemblance matrices were again created from Bray-Curtis distances of both the abundance taxa data and the overall-transformed p/a taxa data (Bray-Curtis similarity, [35]). To test significant difference among years PERMANOVAs [39] were run with the fixed factor "year". For a sharper visualization of temporal variation in community structure, distances between centroids were obtained from the resemblance matrices using "Year" as the grouping factor and visualized with 2D and 3D MDS plots. 3D plots were used to better interpret the direction of vectors in 2D plots and were not included as figures in the current manuscript. Any similar groupings of the centroidal "years" were found with a hierarchical cluster analysis (CLUSTER; [40]). The average sample taxa abundance and presence/absence from each year were calculated, and a BVSTEP Best analysis was ran from the Bray-Curtis resemblance matrix to find the subset of taxa that best explained the community structure variance within centroid data sets (BVSTEP, [37]).

Results

Data collection and isolation

A total of 390 questionnaires were collected from 2007 to 2014 (Table 1). Questionnaire counts per year ranged from 12 to 84, and significantly decreased over time (R^2 = 0.78, P < 0.05). Over the 8 years, 71 (97%) of the 72 target taxa present on the STE questionnaire were sighted (all taxa excluding the Manta Ray).

Preliminary treatment and analysis

According to the RELATE test, years 2007 (ρ_s = 0.162, N = 6, P = 0.007), 2008 (ρ_s = 0.157, N = 2, P = 0.003), 2009 (ρ_s = 0.111, N = 45, P = 0.046), and 2012 (ρ_s = 0.217, N = 9, P = 0.01) showed a significant relationship between taxa abundance and dive parameters (Table 2; Appendix Table 1). In all other years there was no significant relationship (Table 2; Appendix Table 1). The DistLM of taxa abundance data in 2007 showed a significant relationship (P <0.05) with all 6 diving parameters: date (PY), maximum depth (MD), depth where they spent most of their time (AD), temperature (T), dive duration (RTD), and hour (FD)(Table 2; Appendix Table 2). In 2008 all the diving parameters except FD were significantly correlated to the taxa abundance (P < 0.05; Table 2; Appendix Table 2). PY, AD, and FD were significantly related to taxa abundance in 2009 (P < 0.05; Table 2; Appendix Table 2). In 2012 only PY and T dive parameters were significantly related to the community structure (P < 0.05; Table 2; Appendix Table 2).

Table 2. SS Thistlegorm dive parameters with significant ($p<0.05$) sequential DistLM test to community structure by year for abundance and presence/absence data**.** PY as percentage of year, MD as maximum depth, AD as depth at most time spent, T as temperature, RTD as dive duration, and FD as fraction of the day.

Year	Abundance	Presence/Absence
2007	PY, MD, AD, T, RTD, FD	PY, AD, T, RTD, FD
2008	PY, MD, AD, T, RTD	PY, MD, T, RTD
2009	PY, AD, FD	
2010		
2011		
2012	PY, T	PY, T, RTD
2013		
2014		

Regarding taxa p/a data, the Relate results indicated that in 2007 (ρ_s = 0.18, N = 1, P = 0.002), 2008 (ρ_s = 0.136, N = 13, P = 0.014), and 2012 (ρ_s = 0.209, N = 4, P = 0.005) there was a significant relationship between the community structure and dive parameters (Table 2, Appendix Table 1). The DistLM from 2007 revealed that all dive parameters except MD had significant relationships to the taxa p/a data ($P < 0.05$; Table 2, Appendix Table 3). In 2008 PY, MD, T, and RTD were significant ($P < 0.05$; Table 2, Appendix Table 3), while only PY, T and RTD resulted significant in 2012 ($P < 0.05$).

Aggregated diving parameters showed significant differences among years (PERMANOVA, F=9.25, df = 7, P = 0.001). PY, MD, AD, T and RTD all differed significantly among years ($P < 0.05$). FD was homogenous among years.

Of the 7 validation trial dives, the average mean similarity index score was 98.8%, the average mean accuracy score was 57.1% and ranged from 40.4 to 70.0%, and the mean average CAR of 73.8%.

Temporal Analysis

According to the PERMANOVA from both the taxa abundance and p/a data, the community structure was significantly different between each pair of years (F = 4.88, df = 7, P = 0.001 and F = 4.88, df = 7, P = 0.001 respectively). The variation in community structure of taxon abundance and p/a through

years 2007-2014 was represented as an MDS (Figs. 2a and 3a). Given the high stress value (> 0.3) of these MDS plots, further centroidal MDS plots of both taxa data sets were generated to have a clearer, even if more general, visualization highlighting the similar groupings of years within their respective distances resulting from the hierarchical cluster analysis (Figs. 2b and 3b).

Figure 2. a) MDS plot of the SS Thistlegorm taxa abundance community structure over 8 years. Each point represents an individual questionnaire while the different shapes/colors indicate the different years. b) Centroidal MDS of SS Thistlegorm taxa abundance community structure variation over 8 years with overlayed vectors of aggregated best taxa and the indicated trajectories from 2007-2014. Each point represents the central location of the groups “Year”. The green circles surround the indicated groups are from the hierarchical cluster analysis.

Figure 3. a) MDS plot of the SS Thistlegorm taxa presence/absence community structure over 8 years. Each point represents an individual questionnaire while the different shapes/colors indicate the different years. b) Centroidal MDS of SS Thistlegorm taxa presence/absence community structure variation over 8 years with overlayed vectors of aggregated best taxa and the indicated trajectories from 2007-2014. Each point represents the central location of the groups “Year”. The green circles surround the indicated groups are from the hierarchical cluster analysis

The subset of taxa that best explained the distances between yearly centroids of community structure for abundance data were the Soft Tree Coral, Giant Moray, Red Sea Clownfish, Napoleon Wrasse, and Caranxes. The best subset of taxa explaining the distances between yearly centroids of community structure for the p/a data were the Soft Tree Coral, Giant Moray, Squirrel Fish, Humpback Batfish, and Caranxes. Tables 3 and 4 show the relative abundance and SF% of the best taxa through the 8 years.

Table 3. Yearly relative abundance values of best species subset from centroid aggregated taxa abundance data.

Year	Soft Tree Coral	Giant Moray	Red Sea Clownfish	Napoleon Wrasse	Caranxes
2007	77	48	64	26	117
2008	69	26	98	27	94
2009	41	78	80	59	125
2010	98	93	44	37	154
2011	61	63	49	47	114
2012	31	121	35	55	93
2013	50	67	17	50	8
2014	154	62	31	0	115

Table 4. Yearly sighting frequencies of best species subset from centroid aggregated p/a data.

Year	Soft Tree Coral	Giant Moray	Squirrelfish	Humpback Batfish	Caranxes
2007	46%	38%	33%	70%	55%
2008	53%	21%	16%	61%	53%
2009	34%	61%	24%	56%	71%
2010	54%	70%	42%	86%	75%
2011	44%	47%	38%	61%	65%
2012	28%	66%	52%	62%	52%
2013	33%	67%	50%	33%	8%
2014	92%	54%	8%	70%	69%

Discussion

We report here the first investigation on the temporal community trends of one of the most famous and historically significant wrecks worldwide [23] and one of the most visited dive sites in the Red Sea [22]. Scientific literature concerning long-term (more than 5 years) temporal trends and monitoring of community structure on wreck and/or artificial reefs is scarce [32]. There is extensive interest, however, in the role of artificial reefs, (including wrecks and scuttled ships) as a conservation tool, potentially serving as a compensatory habitat for anthropogenically damaged natural reefs [41, 42,43], and as possible refugia assisting corals in the colonization of cooler waters in response to ocean warming [32]. Much of the work regarding artificial coral reefs examine differences between colonization patterns on various substratum material or recently scuttled ships (less than 3 years) and natural reefs, which are largely short-term studies [41, 42, 43, 45, 46]. The few studies that have

investigated community structure change over the long term (more than 5 years) have primarily been snapshot comparisons between the virgin and mature communities [47, 48] or follow succession patterns of virgin artificial reefs [49].

The present study begins to fill this gap through an 8-year monitoring of a well-established wreck community (i.e. > 80 years since the sinking of the SS Thistlegorm in 1941). Despite the internal fluctuation among years (50% in 2013, 93% in 2007), all target taxa, apart from the Manta Ray, were sighted throughout the duration of this study. These findings indicate a long-term high, if variable, diversity in the biological community at the Thistlegorm and are in line with the results of the monitoring of natural reefs in the region where reef sites far from touristic facilities show higher biodiversity [50]. The Kingston, a 119-year-old shipwreck 10 km east of the Thistlegorm, mimics the surrounding natural reef communities due to its similarities in structural complexity [51]. Oil jetties in Eilat (northern Red Sea) studied as artificial reef proxies, have even higher species richness in fish assemblages than neighboring natural reefs [46] due to increased structural complexity. Studies of artificial reefs and wrecks in other regions throughout the world have shown similarly promising results with respect to high community biodiversity, supporting the findings at the SS Thistlegorm. In the Florida Keys, artificial reef fish communities mirror those of natural reefs in terms of fish numbers and species composition [52] and two thirds of genera from neighboring natural reefs can thrive on sunken shipwrecks within the Caribbean [32]. Even in temperate seas, such as the North Sea along the Belgian Continental Shelf, shipwrecks are described as biodiversity hotspots of hard substrate communities in an otherwise sandy bottom environment while protecting nearby soft-bottom communities from fishing activities [33, 34].

The number of observations collected in this study through recreational citizen science methods decreased in time (Table 1). This is ascribable to the reduction in overall tourism due to political upheaval consuming Egypt during the revolution and the following transitional period of the "Arab Spring" [53,54]. As tourists seek to vacation in more stable and safe countries, participation in citizen science is inherently affected. This temporal non-homogeneity is an evident and

acknowledged limitation of citizen science, and thus it should not outweigh the benefits (e.g., increasing participant education and awareness, reduction of economic and time costs associated with environmental monitoring, and acceptable level of accuracy in data collection) of such a monitoring approach [19, 20, 50, 55].

Diving parameters were significantly corelated with the taxa data in some of the years. PY was the most frequently related dive parameter, appearing in all years with a significant relationship to diving parameters for both the taxa abundance and taxa p/a data sets (Table 2). The yearly average PY spans about half a year, ranging from early April in 2014 to late September in 2009, with little internal variation (Appendix Table 4). Some of the variation in community structure over time in this study could thus be due to the differing seasonal environmental conditions in which questionnaires were collected. Total macroalgal biomass and community structure on coral reefs are strongly linked to seasonality and temperature variation in the Red Sea [55], where variation in benthic communities also affect the distribution of fish species [57]. Species richness, diversity, and evenness of reef fish assemblages vary significantly between summer and winter in the Red Sea [58], and these seasonal variations may be mirrored on wrecks and artificial reefs close to natural coral reefs.

Temperature, the next most linked dive parameter (Table 2) is generally influenced by both seasonality and depth. In this study, T is more likely a function of the seasonal variation because the range in average depth (AD) and Maximum Depth (MD) throughout the years is quite small (19-22 m and 25-28 m respectively) (Appendix Table 4). In fact, coral species that dominate shallows depths tend to occupy more narrow depth ranges while species that can colonize deeper in the water column have wider depth ranges (Chow e[illegible]. 2019), suggesting that the small range of average depth in surveys at the Thistlegorm, even if statistically significant, is trivial in terms of its effect on the community structure variation given the greater depth at which the surveys were conducted. This is especially true when considering non-sessile benthic and/or free-swimming taxa. The yearly average dive duration ranged from 41 to 55 minutes (Appendix Table 4) and presented a significant correlation in 2007 and 2008 for both data sets and in 2012 for the taxa p/a data (Table 2). Dive duration certainly

plays a role in the number and amount of target taxa sighted. In validation trials of the STE project (Mesch[illegible] et al. 2021), volunteer scores of mean accuracies, mean consistency, and mean percent identified are all positively correlated with dive time.

Following the temporal sequence of centroids of taxa abundance (Fig. 2b), no clear trend emerged. Years 2007, 2009, and 2011 cluster around what could be interpreted as a general (even if variable) community composition. The Red Sea Clownfish (*Amphiprion bicinctus*) was overrepresented in 2008 (98 in 2008 *vs* 49-80 in 2007, 2009, and 2011; Table 3). The year 2010 was overrepresented in Soft Tree Coral (*Dendronepthya spp.*) (98 in 2010 *vs* 41-77 in 2007, 2009, and 2011; Table 3), and Giant Moray (*Gymnothorax javanicus*) (93 in 2010 *vs* 48-78 in 2007, 2009, and 2011; Table 3) and underrepresented in Red Sea Clownfish (44 in 2010 *vs* 49-80 in 2007,2009, and 2011; Table 3). In 2012 there was an overrepresentation of Giant Moray (121 in 2012 *vs* 48-78 in 2007, 2009 and 2011; Table 3) and Napoleon Wrasse (*Cheilinus undulatus*) (55 in 2012 *vs* 26-59 in 2007,2009, and 2011; Table 3) with an underrepresentation of Soft Tree Coral (31 in 2012 *vs* 41-77 in 2007, 2009 and 2011; Table 3) and Red Sea Clownfish (35 in 2012 *vs* 49-80 in 2007, 2009 and 2011; Table 3). In 2013 there was an underrepresentation of Caranxes (*Caranx spp.*) (8 in 2013 *vs* 114-125 in 2007, 2009, and 2011; Table 3) and Red Sea Clownfish (17 in 2013 *vs* 49-80 in 2007, 2009, and 2011; Table 3). 2014 was overrepresented by Soft Tree Coral (154 in 2014 *vs* 41-77 in 2007, 2009 and 2011; Table 3) and underrepresented by Red Sea Clownfish (31 in 2014 *vs* 49-80 in 2007, 2009 and 2011; Table 3) and Napoleon Wrasse (0 in 2014 *vs* 26-59 in 2007,2009, and 2011; Table 3).

The p/a centroidal data (Fig 3b) displayed a general cluster in community structure, like the abundance data, but it was larger and represented by the years 2007 through 2012. With respect to this general cluster the year 2013 exhibited an underrepresentation in Humpback batfish (*Platax bativianus*) (33% in 2013 vs 56-86% in 2007-2012; Table 4) and Caranxes (8% in 2013 vs 52-75% in 2007-2012; Table 4). The following year showed a community similar to the general cluster but with an overrepresentation of Soft Tree Coral (92% in 2014 vs 28-54% in 2007-2012; Table 4) and

an underrepresentation of Squirrel Fish (*Holocentrus rufus*) (8% in 2014 *vs* 16-52% in 2007-2012; Table 4).

The taxa abundance data revealed more variance among years than taxa p/a data, and even if the general trend is quite similar, two of the five b[illegible] species best explaining community structure variations among years are different in the analysis of the two data sets (the Red Sea Clownfish and Napoleon Wrasse for p/a data *vs*. the Squirrel Fish and Humpback Batfish for abundance data). This partial inconsistency may depend on the different reliabilities of volunteer collected data between presence/absence and abundance data sets. In fact, the correctness of abundance parameter ratings ranges from 41 to 82 percent in validation trials of the entire STE project (and from 64 to 82 percent in the present analysis) while groups of volunteers show better performances in correctly identifying the presence of taxa [19, 20]. In 7 validation trials from the SS Thistlegorm, the parameters of data quality measurements were in line with previous analyses on coral reef environment types within the STE project [20]. The SS Thistlegorm average mean accuracy scored 57.1 percent within a range of 46 to 70 percent, coinciding with the results from [20] where 94 percent of trials scored a mean accuracy between 40 and 70 percent. From the similarity index (SI) parameter all 7 of the dives analyzed here resulted in high levels of precision (SI, 95% CI lower bound > 75% ≤ 100%), whereas only 0.4 percent of trials in [20] obtained these similarity index scores. Notwithstanding the small number of validation trials ran on the SS Thistlegorm data set with respect to the entire STE project [20], the results highly resemble those of the more rigorous trials analyzed in [20] and can therefore provide enhanced reliability to the data that has been used for evaluating the community structure variance over time at this particular dive site.

In conclusion, there was no clear sequential shift of the SS Thistlegorm community structure over the eight years of monitoring, but some fluctuation around a general cluster characterized by high biodiversity, mainly driven by yearly relative changes in the frequency of a few species. The temporal analysis may have been slightly biased by the different average PY (i.e., seasonality) of collected data, but this bias is likely to be minimal, as the years in which PY was significantly related

to taxa data are mainly included in the general cluster and do not display unusual patterns. The community structure at the SS Thistlegorm showed relative stability over time, making this artificial reef a possible and promising refugia for Red Sea biodiversity. Further investigation on the influence of artificial reefs as an auxiliary tool for human-influenced decline could entail the comparison of multiple wreck sites within the northern Red Sea to nearby natural coral reef dive sites through species abundance analyses between and among groupings of sites, possibly by latitudinal and/or temperature gradients.

Acknowledgements

Special thanks go to all the divers who made this study possible. STE project was funded by Project AWARE Foundation, ASTOI Association, Ministry of Tourism of the Arab Republic of Egypt, Settemari S.p.A Tour Operator, Scuba Nitrox Safety International, Viaggio nel Blu Diving Center.

Reference List

1. Bellwood DR, Hughes TP. Regional-scale assembly rules and biodiversity of coral reefs. Science. 2001 May 25;292(5521):1532–5.

2. Woodhead AJ, Hicks CC, Norström AV, Williams GJ, Graham NAJ. Coral reef ecosystem services in the Anthropocene. Fox C, editor. Functional Ecology. 2019 Mar 28;33(6).

3. Aronson RB, Macintyre IG, Lewis SA, Hilbun NL. Emergent zonation and geographic convergence of coral reefs. Ecology. 2005 Oct;86(10):2586–600.

4. Carpenter KE, Abrar M, Aeby G, Aronson RB, Banks S, Bruckner A, et al. One-third of reef-building corals face elevated extinction risk from climate change and local impacts. Science [Internet]. 2008 Jul 25;321(5888):560–3.

5. Kubicek A, Breckling B, Hoegh-Guldberg O, Reuter H. Climate change drives trait-shifts in coral reef communities. Scientific Reports [Internet]. 2019 Mar 6;9(1):1–10.

6. Bindoff NL, Cheung WW, Kairo JG, Arístegui J, Guinder VA, Hallberg R, et al. Changing ocean, marine ecosystems, and dependent communities. IPCC special report on the ocean and cryosphere in a changing climate. 2019:477-587.

7. Fine M, Cinar M, Voolstra CR, Safa A, Rinkevich B, Laffoley D, et al. Coral reefs of the Red Sea — Challenges and potential solutions. Regional Studies in Marine Science. 2019 Jan;25:100498.

8. DiBattista JD, Roberts MB, Bouwmeester J, Bowen BW, Coker DJ, Lozano-Cortés DF, et al. A review of contemporary patterns of endemism for shallow water reef fauna in the Red Sea. Journal of Biogeography. 2015 Nov 3;43(3):423–39.

9. Kleinhaus K, Al-Sawalmih A, Barshis DJ, Genin A, Grace LN, Hoegh-Guldberg O, et al. Science, diplomacy, and the Red Sea's unique coral reef: it's time for action. Frontiers in Marine Science. 2020 Feb 26.

10. Riegl BM, Bruckner AW, Rowlands GP, Purkis SJ, Renaud P. Red Sea coral reef trajectories over 2 decades suggest increasing community homogenization and decline in coral size. Roberts JM, editor. PLoS ONE. 2012 May 31;7(5):e38396.

11. Tesfamichael D, Pitcher TJ. Multidisciplinary evaluation of the sustainability of Red Sea fisheries using Rapfish. Fisheries Research. 2006 May 1;78(2-3):227-35.

12. Gladstone W, Curley B, Shokri MR. Environmental impacts of tourism in the Gulf and the Red Sea. Marine Pollution Bulletin. 2013 Jul;72(2):375–88.

13. Berumen ML, Roberts MB, Sinclair-Taylor TH, DiBattista JD, Saenz-Agudelo P, Isari S, et al. Fishes and Connectivity of Red Sea Coral Reefs. Coral Reefs of the Red Sea. 2019;11:157–79.

14. Sauermann H, Franzoni C. Crowd science user contribution patterns and their implications. Proceedings of the National Academy of Sciences. 2015 Jan 5;112(3):679–84.

15. Silvertown J. A new dawn for citizen science. Trends in Ecology & Evolution. 2009 Sep;24(9):467–71.

16. Kobori H, Dickinson JL, Washitani I, Sakurai R, Amano T, Komatsu N, et al. Citizen science: a new approach to advance ecology, education, and conservation. Ecological Research [Internet]. 2015 Nov 17;31(1):1–19.

17. Keough HL, Blahna DJ. Achieving integrative, collaborative ecosystem management. Conservation Biology. 2006 Oct;20(5):1373–82.

18. Whitelaw G, Vaughan H, Craig B, Atkinson D. Establishing the Canadian Community Monitoring Network. Environmental Monitoring and Assessment. 2003;88(1/3):409–18.

19. Branchini S, Meschini M, Covi C, Piccinetti C, Zaccanti F, Goffredo S. Participating in a Citizen Science Monitoring Program: Implications for Environmental Education. Bernardi G, editor. PLOS ONE. 2015 Jul 22;10(7):e0131812.

20. Meschini M, Machado Toffolo M, Marchini C, Caroselli E, Prada F, Mancuso A, et al. Reliability of Data Collected by Volunteers: A Nine-Year Citizen Science Study in the Red Sea. Frontiers in Ecology and Evolution. 2021 Jun 24;9.

21. El Gohary G. Wet landscape: using sculptures to form underwater landscape. Democratic Transition and Sustainable Communities. 2013 Nov 6:351.

22. Motawea Hussein SHAIKHON A. Managing underwater heritage in Egypt. International Journal of Advanced Studies in World Archaeology. 2019 Jun 1;2(1):1–13.

23. Brown S, Henderson J, Mustard A, Postons M. Diving the Thistlegorm: the ultimate guide to a world war II shipwreck. S.L.: Dived Up Publications; 2021.

24. Edney J, Boyd WE. Diving under the radar: divers and submerged aircraft. Journal of Heritage Tourism. 2020 May 28;16(1):100–17.

25. Maynard JA. Severe anchor damage to Lobophyllia variegata colonies on the Fujikawa Maru, Truk Lagoon, Micronesia. Coral Reefs. 2008 Oct 31;27(2):273–3.

26. Bombace G. Artificial reefs in the Mediterranean Sea. Bulletin of marine Science. 1989 Mar 1;44(2):1023-32

27. Seixas LB, Barreto NR, Santos LN dos. Artificial reefs for marine and freshwater fish management in Brazil: Researchers profile and academic production over the 1990-2010 period. Oecologia Australis. 2013 Sep;17(3):374–85.

28. Tessier A, Dalias N, Lenfant P. Expectations of professional and recreational users of artificial reefs in the Gulf of Lion, France. Journal of Applied Ichthyology. 2015 Dec;31:60–73.

29. Polak O, Shashar N. Can a small artificial reef reduce diving pressure from a natural coral reef? Lessons learned from Eilat, Red Sea. Ocean & Coastal Management. 2012 Jan;55:94–100.

30. Pinheiro HT, Bernardi G, Simon T, Joyeux J-C, Macieira RM, Gasparini JL, et al. Island biogeography of marine organisms. Nature [Internet]. 2017;549(7670):82–5.

31. Macarthur RH, Wilson EO. The theory of island biogeography. Princeton, N.J: Princeton University Press, Tr; 1967.

32. Asner GP, Giardina SF, Balzotti C, Drury C, Hopson S, Martin RE. Are Sunken Warships Biodiversity Havens for Corals? Diversity. 2022 Feb 16;14(2):139.

33. Lengkeek W, Coolen JW, Gittenberger A, Schrieken N. Ecological relevance of shipwrecks in the North Sea. Nederlandse Faunistische Mededelingen. 2013 Jan 1;41:49-57.

34. Mallefet J, Zintzen V, Massin C, Norro A, Vincx M, De Maersschalck V, Steyaert M, Degraer S, Cattrijsse A, Vanden Berghe E. Belgian shipwreck: hotspots for marine biodiversity (BEWREMABI). Final Scientific Report. Belgian Science Policy. 155pp. 2008 May.

35. Shepard RN. The analysis of proximities: Multidimensional scaling with an unknown distance function. II. Psychometrika. 1962 Sep;27(3):219-46.

36. Kruskal JB. Multidimensional scaling by optimizing goodness of fit to a nonmetric hypothesis. Psychometrika. 1964 Mar;29(1):1-27.

37. Clarke K, Ainsworth M. A method of linking multivariate community structure to environmental variables. Marine Ecology Progress Series. 1993;92:205–19.

38. Clarke KR, Gorley RN. Primer v6 : user manual - tutorial. Plymouth Plymouth Marine Laboratory; 2006.

39. McArdle BH, Anderson MJ. Fitting multivariate models to community data: a comment on distance-based redundancy analysis. Ecology. 2001 Jan;82(1):290–7.

40. Kaufman L, Rousseeuw PJ. Finding Groups in Data [Internet]. Kaufman L, Rousseeuw PJ, editors. Wiley Series in Probability and Statistics. Hoboken, NJ, USA: John Wiley & Sons, Inc.; 1990.

41. Higgins E, Scheibling RE, Desilets KM, Metaxas A. Benthic community succession on artificial and natural coral reefs in the northern Gulf of Aqaba, Red Sea. Ferse SCA, editor. PLOS ONE. 2019 Feb 27;14(2):e0212842.

42. Perkol-Finkel S, Benayahu Y. Community structure of stony and soft corals on vertical unplanned artificial reefs in Eilat (Red Sea): comparison to natural reefs. Coral Reefs. 2004 May 14;23(2).

43. Walker SJ, Schlacher TA. Limited habitat and conservation value of a young artificial reef. Biodiversity and Conservation. 2014 Jan 4;23(2):433–47.

44. Kumar JY, Geetha S, Raghunathan C, Venkataraman K. An Assessment of Faunal Diversity and its Conservation in Shipwrecks in Indian Seas. Marine Faunal Diversity in India 2015 Jan 1 (pp. 441-452). Academic Press.

45. Monchanin C, Mehrotra R, Haskin E, Scott CM, Urgell Plaza P, Allchurch A, et al. Contrasting coral community structures between natural and artificial substrates at Koh Tao, Gulf of Thailand. Marine Environmental Research. 2021 Dec;172:105505.

46. Rilov G, Benayahu Y. Fish assemblage on natural versus vertical artificial reefs: the rehabilitation perspective. Marine Biology. 2000 Jun 16;136(5):931–42.

47. Abelson A, Shlesinger Y. Comparison of the development of coral and fish communities on rock-aggregated artificial reefs in Eilat, Red Sea. ICES Journal of Marine Science. 2002 Oct;59:S122–6.

48. Kotb M. Coral colonization and fish assemblage on an artificial reef off Hurghada, Red Sea, Egypt. Egyptian Journal of Aquatic Biology and Fisheries. 2013 Dec 1;17(4):71-81.

49. Nicoletti L, Marzialetti S, Paganelli D, Ardizzone GD. Long-term changes in a benthic assemblage associated with artificial reefs. Hydrobiologia. 2007 Apr;580(1):233–40.

50. Branchini S, Pensa F, Neri P, Tonucci BM, Mattielli L, Collavo A, et al. Using a citizen science program to monitor coral reef biodiversity through space and time. Biodiversity and Conservation. 2015 Oct 7;24(2):319–36.

51. Perkol-Finkel S, Shashar N, Benayahu Y. Can artificial reefs mimic natural reef communities? The roles of structural features and age. Marine Environmental Research. 2006 Mar;61(2):121–35.

52. Stone RB, Pratt HL, Parker Jr RO, Davis GE. A comparison of fish populations on an artificial and natural reef in the Florida Keys. Mar. Fish. Rev. 1979 Sep 1;41(9):1-1.

53. Esmail HA. Impact of Terrorism and instability on the tourism industry in Egypt and Tunisia after Revolution. The Business & Management Review. 2016 Jun 1;7(5):469.

54. Wendt JA. Comparison of the impact of the Arab Spring and terrorist attacks on the decline in tourism in Egypt and Tunisia (2010-2015). GeoJournal of Tourism and Geosites. 2019 Dec 31;27(4):1367–76.

55. Goffredo S, Pensa F, Neri P, Orlandi A, Gagliardi MS, Velardi A, et al. Unite research with what citizens do for fun: “recreational monitoring” of marine biodiversity. Ecological Applications. 2010 Dec;20(8):2170–87.

56. Ateweberhan M, Bruggemann JH, Breeman AM. Effects of extreme seasonality on community structure and functional group dynamics of coral reef algae in the southern Red Sea (Eritrea). Coral Reefs. 2006 Apr 14;25(3):391–406.

57. Eyal G, Tamir R, Kramer N, Eyal-Shaham L, Loya Y. The red sea: Israel. InMesophotic coral ecosystems 2019 (pp. 199-214). Springer, Cham.

58. Megdadi JM, Khalaf MA, Al-Horani FA, Manasrah RS. Community structure of coral reef fishes in relation to habitat and depth in the northern Gulf of Aqaba, Red Sea. Fresenius Environ. Bull. 2017 Jan 1;26:1824-34.

59. Chow GSE, Chan YKS, Jain SS, Huang D. Light limitation selects for depth generalists in urbanized reef coral communities. Marine Environmental Research. 2019 May;147:101–1.

Figure 1

Click here to access/download;Figure;Fig1.tif

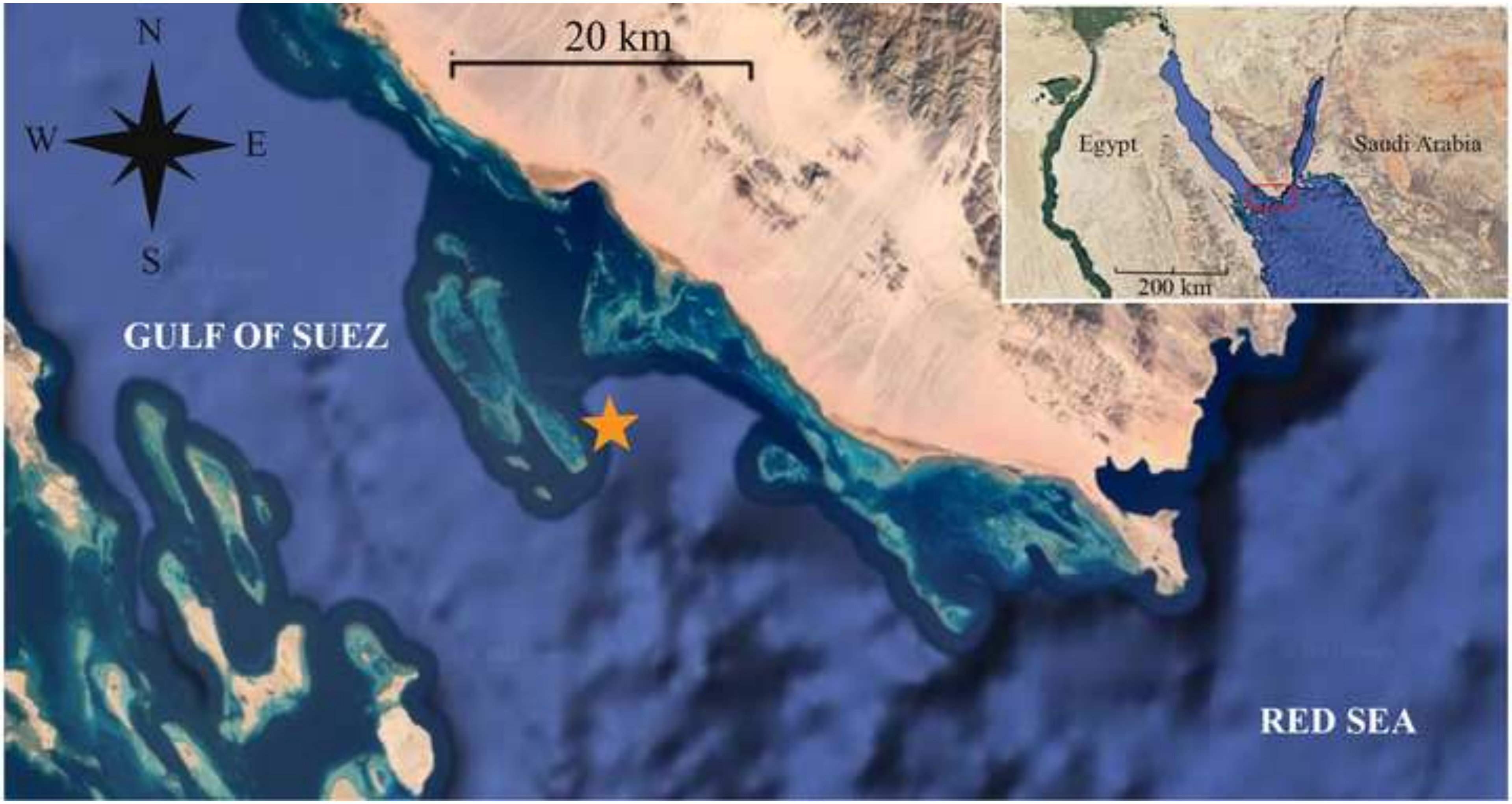

Figure 2

Click here to access/download;Figure;Fig2.tif

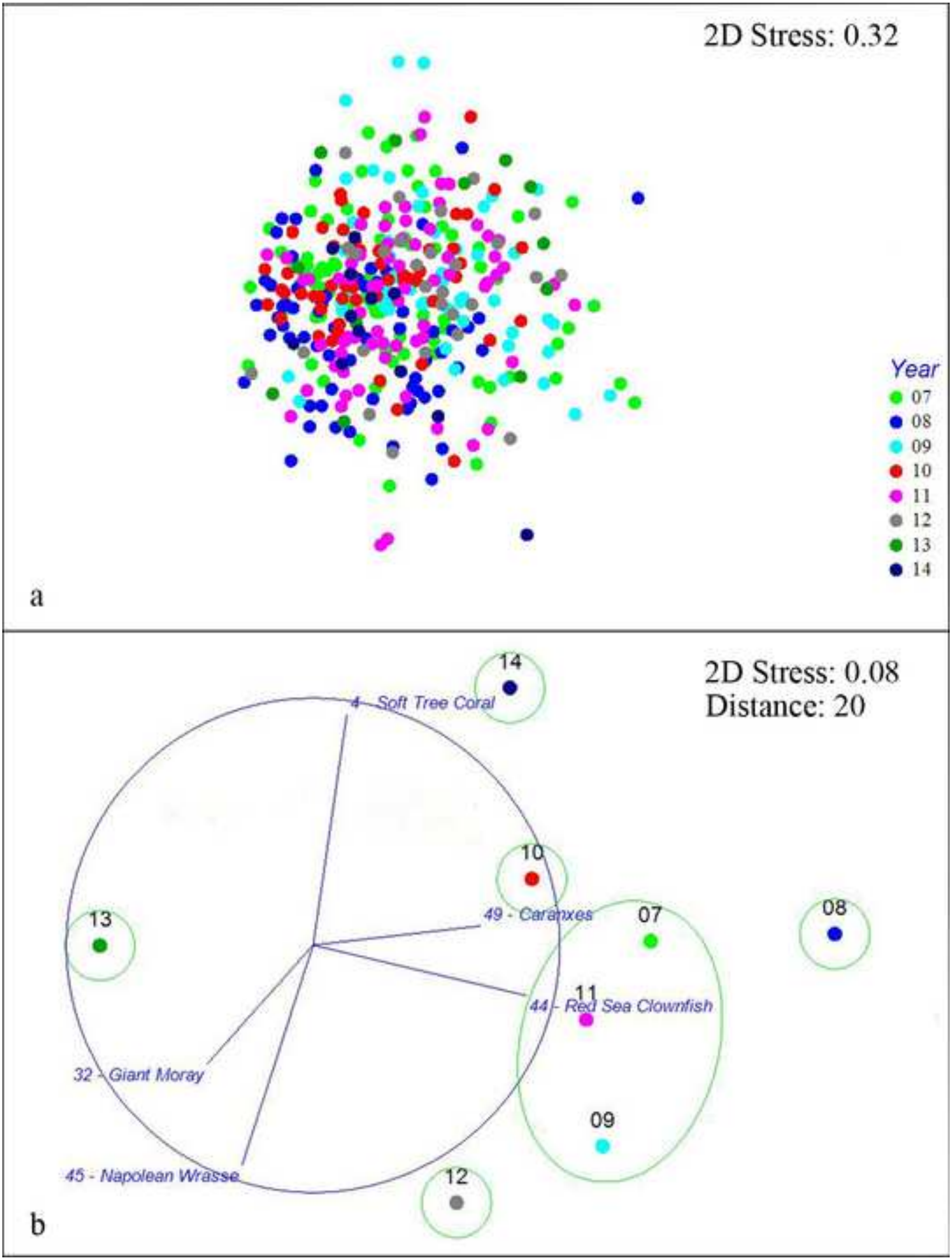

Figure 3

Click here to access/download;Figure;Fig3.tif

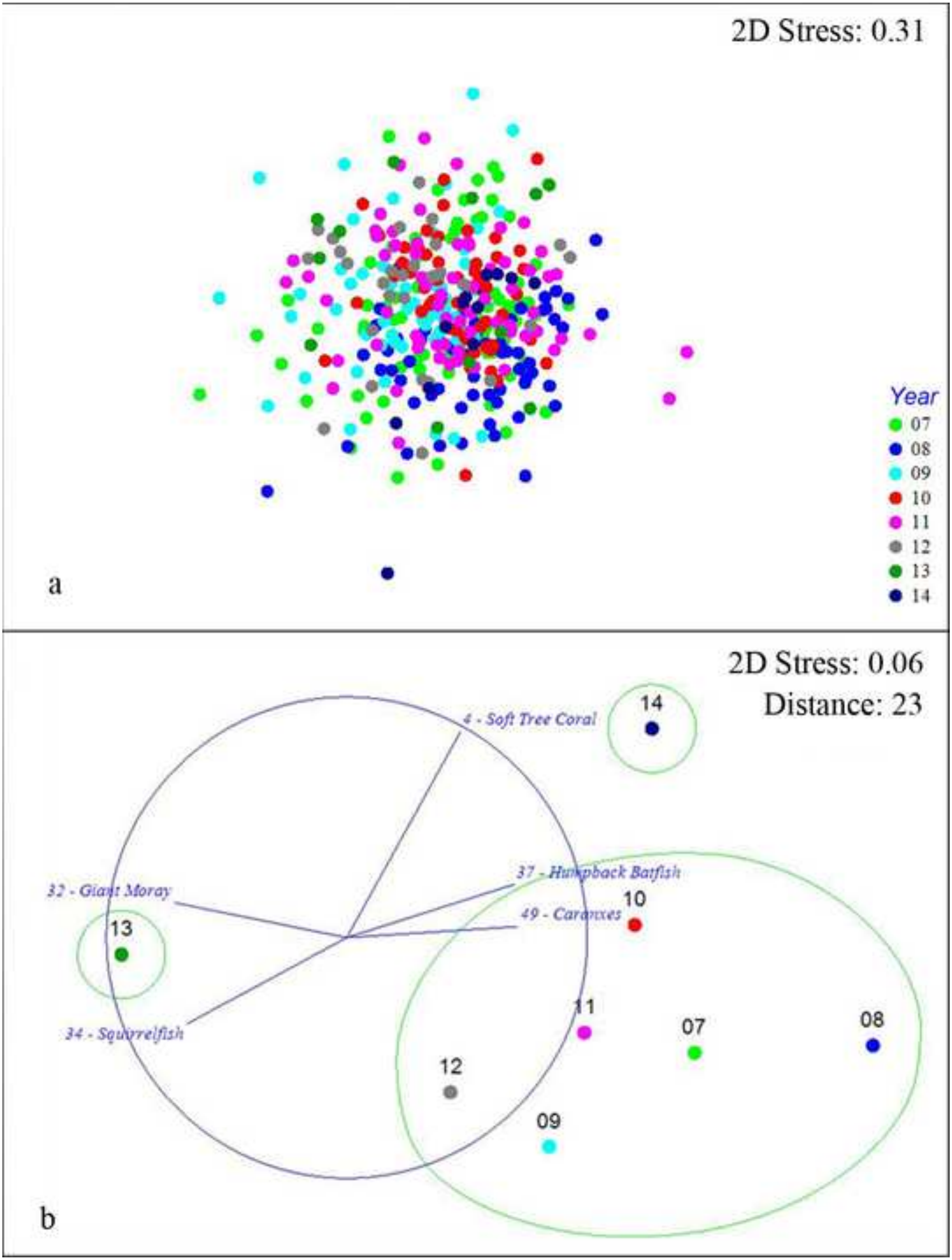

Click here to access/download
Supporting Information
supplementary.docx
